# Genomic islands of divergence in the Yellow Tang and the Brushtail Tang Surgeonfishes

**DOI:** 10.1002/ece3.4417

**Published:** 2018-08-02

**Authors:** Giacomo Bernardi, Peter Nelson, Michelle Paddack, John Rulmal, Nicole Crane

**Affiliations:** ^1^ Department of Ecology and Evolutionary Biology University of California Santa Cruz Santa Cruz California; ^2^ H. T. Harvey & Associates Los Gatos California; ^3^ Santa Barbara City College Santa Barbara California; ^4^ Ulithi Falalop Community Action Program Yap Federated States of Micronesia; ^5^ Department of Biology Cabrillo College Aptos California

**Keywords:** outlier loci, population genomics, Species delimitations, *Zebrasoma flavescens*, *Zebrasoma scopas*

## Abstract

The current ease of obtaining thousands of molecular markers challenges the notion that full phylogenetic concordance, as proposed by phylogenetic species concepts, is a requirement for defining species delimitations. Indeed, the presence of genomic islands of divergence, which may be the cause, or in some cases the consequence, of speciation, precludes concordance. Here, we explore this issue using thousands of RAD markers on two sister species of surgeonfishes (Teleostei: Acanthuridae), *Zebrasoma flavescens* and *Z. scopas,* and several populations within each species. Species are readily distinguished based on their colors (solid yellow and solid brown, respectively), yet populations and species are neither distinguishable using mitochondrial markers (cytochrome c oxidase 1), nor using 5193 SNPs (pairwise Φst = 0.034). In contrast, when using outlier loci, some of them presumably under selection, species delimitations, and strong population structure follow recognized taxonomic positions (pairwise Φst = 0.326). Species and population delimitation differences based on neutral and selected markers are likely due to local adaptation, thus being consistent with the idea that these genomic islands of divergence arose as a consequence of isolation. These findings, which are not unique, raise the question of a potentially important pathway of divergence based on local adaptation that is only evident when looking at thousands of loci.

## 1 INTRODUCTION

Defining species has important theoretical and practical implications, yet no unified concept and approach have emerged (De Queiroz, 1998, 2007; Hausdorf, 2011). With the advent of genetic markers, debates over species concepts resurfaced (Noor & Feder, 2006). However, for a long time, only few markers (first strictly mitochondrial, then nuclear) were used, and the tenets of phylogenetic species concepts, where a congruence of multiple molecular markers is central for species delimitations (Cracraft, 1983; Moritz, 1994), could still be easily tested and achieved (e.g., Bernardi, Sordino, & Powers, 1993). The use of few markers to reconstruct phylogenetic relationships, however, missed important information at the level of the entire genome. Of late, phylogenomics and population genomics brought again to the forefront the issues of congruence, but now among hundreds of markers (Leaché, Fujita, Minin, & Bouckaert, 2014; Longo & Bernardi, 2015; Rannala, 2015; Tariel, Longo, & Bernardi, 2016; Wagner et al., 2013). Early studies showed that genomes are a complex mosaic, with large stretches of neutral and selected regions that display very different dynamics (Turner, Hahn, & Nuzhdin, 2005). Genomic regions under selection or under differential recombination dynamics were termed “genomic islands of divergence” (Berner & Roesti, 2017; Huang, Huang, Huang, & Liao, 2017; Ma et al., 2017; Wolf & Ellegren, 2016). In some cases, these were shown to play an important role both in the engine of speciation and in maintaining species boundaries, thus called genomic islands of speciation (Cruickshank & Hahn, 2014; Farré, Micheletti, & Ruiz‐Herrera, 2013; Marques et al., 2016; Noor & Bennett, 2009; Nosil, 2012; Nosil & Feder, 2012; Turner et al., 2005). Genomic islands of divergence exhibit higher‐than‐expected fixation indexes (Fsts) and may be identified by analyses of outlier loci (Egan, Nosil, & Funk, 2008; Via, Conte, Mason‐Foley, & Mills, 2012). Systems to explore these issues include taxa in the early stages of speciation, where an analysis of pairwise genomic differentiation (Fst) is powerful (Marques et al., 2016). Instead, populations that have been physically separated in different environments and experience little gene flow may exhibit local adaptation, which also results in regions under selection (Bernardi, Azzurro, Golani, & Miller, 2016; Gaither et al., 2015; Sandoval‐Castillo, Robinson, Hart, Strain, & Beheregaray, 2018). In this case, the genomic islands of divergence are likely to be a consequence of physical separation and speciation rather than their cause.

Here, we explore the use of neutral and outlier loci in two closely related surgeonfishes, *Zebrasoma flavescens* and *Z. scopas*. These mostly allopatric species have been shown to be poorly genetically differentiated, a potential hallmark of incipient speciation (Bernardi, 2013; Coyne & Orr, 2004).

**Figure 1:**
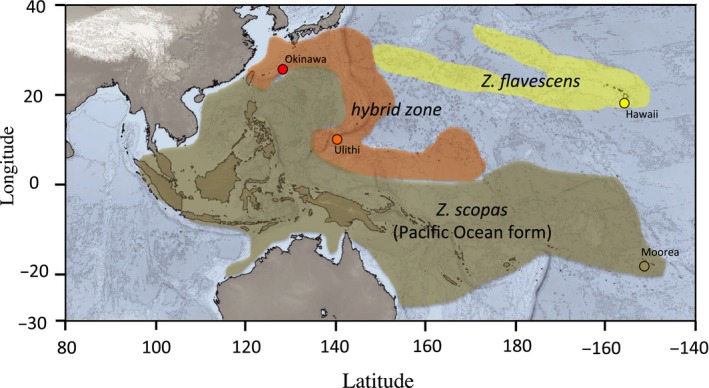
Geographic distribution of *Zebrasoma flavescens* (yellow shaded area), *Zebrasoma scopas* (brown shaded area), and their hybrid contact zone (orange shaded area). Sampling locations are Okinawa (Japan), Hawaii (Kona, USA), Ulithi (Federated States of Micronesia), Moorea (French Polynesia) [Colour figure can be viewed at http://wileyonlinelibrary.com]

Surgeonfishes (*Acanthuridae)* include six genera of herbivorous reef fishes that broadcast spawn, usually in large groups (Kuiter & Debelius, 2001; Randall, 2001). One genus, *Zebrasoma,* includes seven species, with two very closely related taxa, the yellow tang, *Z. flavescens* (one of the most popular fish of the pet trade)*,* and the brushtail tang, *Z. scopas* (Randall, 1955, 2001; Sorenson, Santini, Carnevale, & Alfaro, 2013). These two species have different geographic distributions, with a wide Indo‐Pacific distribution for *Z. scopas,* and a restricted range for *Z. flavescens* (North Pacific including Hawaiian islands) (Figure 1). Regions of overlap between the species are present in the northeastern range of *Z. scopas* and the western range of *Z. flavescens* centered at the Marianas, Marshall, and Micronesian islands, and including southern Japan (Figure 1). There, hybrids between the two species have been reported (Kuiter & Debelius, 2001; Randall, 2001).

**Figure 2:**
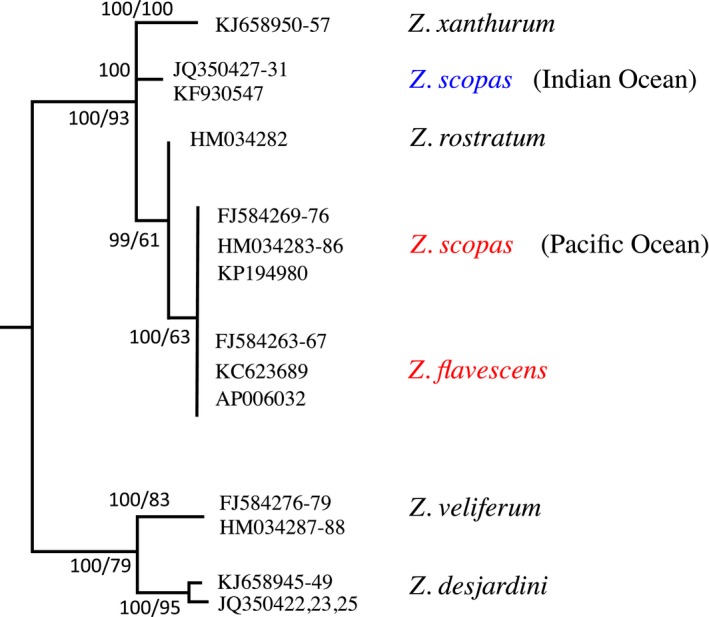
Phylogenetic relationships of the surgeonfish genus *Zebrasoma* based on the barcoding gene cytochrome c oxidase 1 (CO1). Neighbor‐joining (NJ) and maximum likelihood (ML) method recovered the same topology (NJ is shown here) results of 1,000 bootstrap replicates as shown as nodal supports near the corresponding nodes when above 50% (NJ number first, ML number second). *Zebrasoma scopas* was found to be paraphyletic with an Indian Ocean form (blue) and a Pacific Ocean form (red). Species investigated in this study are shown in red. Taxa numbers correspond to GenBank accession numbers, and tree is rooted with the closely related *Paracanthurus hepatus* [Colour figure can be viewed at http://wileyonlinelibrary.com]

Limited published genetic work has shed some light on the evolution of *Z. scopas* and *Z. flavescens*. Population genetics of *Z. flavescens* based on mitochondrial and microsatellite markers showed that population structure was present across the species range (Eble et al., 2011). Phylogenetic studies based on the universal barcoding region (mitochondrial cytochrome c oxidase 1, CO1) have highlighted two main findings: First, the species *Zebrasoma scopas* most likely corresponds to two paraphyletic species restricted to either the Indian Ocean or the Pacific Ocean (Figure 2) (Hubert et al., 2012); and second, CO1 markers could not separate *Z. scopas* (the Pacific form) and its closest relative *Z. flavescens* (Figure 2) (Steinke, Zemlak, & Hebert, 2009). This lack of species delimitation was interpreted as either a case of incomplete lineage sorting or a case of species oversplitting (Steinke et al., 2009).

The original *Z. scopas* was described from the Pacific, and this genetic group is therefore most likely to retain the original name when a revision of the genus is published (Bauchot & Randall, 1996). In this manuscript, we will refer to *Z. scopas* as the Pacific clade as shown in Figure 2. It is important that this study focuses on the monophyletic cluster that includes *Z. flavescens* and the Pacific form of *Z. scopas*. It is not intended to examine the taxonomic status of the Indian Ocean form of *Z. scopas*. In fact, this latter species is not sister to the *Z. flavescens–Z. scopas* (Pacific form) cluster (Figure 2) and has therefore no bearing on our study.

Taking into account the lack of resolution at the CO1 sequence level, the presence of hybrid forms, and geographic range overlaps, we considered the *Zebrasoma scopas/flavescens* system ideal to study questions pertaining to understanding the different dynamics of genomic regions. We used hundreds of RAD‐seq markers, both neutral and under selection (outlier loci) for the two species, *Z. flavescens* and *Z. scopas*, as well as for different populations of *Z. scopas,* as a means to internally compare the level of differentiation between species and among populations. In particular, our goals were first to determine whether genomic regions of divergence were present, and in the affirmative, if there was a signature associated with past or present speciation events in this group of fishes.

## 2 MATERIALS AND METHODS

### 2.1 Mitochondrial phylogenetic reconstructions

In order to illustrate the taxonomic positions of *Zebrasoma scopas* and *Z. flavescens* in Introduction of this manuscript*,* we reconstructed a phylogeny of the genus *Zebrasoma* based on published mitochondrial barcoding sequences (cytochrome c oxidase 1). We obtained all available *Zebrasoma* sequences from GenBank and used sequences of *Paracanthurus hepatus* as an outgroup, following published work (Sorenson et al., 2013). Phylogenetic reconstructions were performed based on the neighbor‐joining method generated in R (R Core Team, 2013), with the ape package (Paradis, Claude, & Strimmer, 2004) using Kimura‐2 parameter substitution models, and a maximum likelihood method implemented in GARLI (Zwickl, 2006). Node support was obtained using 1000 bootstrap replicates and retaining values that support nodes in more than 50% of the bootstrap replicates.

### 2.2 Sampling

Samples of adult fishes were collected between July 2013 and August 2017 while free diving using pole spears (JBL, Oceanside, California, USA) (Figure 1). We first sampled areas where no hybrids are recorded. Ten *Zebrasoma flavescens* were collected in Kona, Hawaii, a region where *Z. scopas* is absent. Ten *Z. scopas* were collected from Moorea, French Polynesia, where *Z. flavescens* is absent (and as geographically distant as possible from a potential introgression from the Indian Ocean clade). We then sampled areas within the hybrid zone (Figure 1). Nine *Z. scopas* were collected in Ulithi, Federated States of Micronesia, a region where *Z. flavescens* is present, but in very low numbers. There, five samples identified as *Z. flavescens* were collected. The additional three and two samples, collected in Ulithi, and Okinawa, Japan, respectively, were identified in the field as having intermediate coloration patterns, possibly matching what an expected hybrid would look like (Randall, 2001). Therefore, the samples used in this study corresponded to 19 individuals identified as *Z. scopas,* 15 individuals identified as *Z. flavescens,* and five individuals identified as hybrids for a total of 39 samples.

### 2.3 DNA extractions and RAD libraries

Fin clip tissue samples were stored in 95% ethanol and DNA was extracted using DNeasy Blood & Tissue Kits (Qiagen) according to the manufacturer's protocol. We constructed RAD libraries using a variation of the original protocol with restriction enzyme SbfI (Baird et al., 2008; Longo & Bernardi, 2015; Miller, Dunham, Amores, Cresko, & Johnson, 2007; Miller et al., 2012; Omar et al., 2016). Individually barcoded samples were sequenced on an Illumina HiSeq 2500 at the Vincent J. Coates Genomics Sequencing Laboratory at UC Berkeley.

### 2.4 Quality filtering and marker discovery

Raw 100‐bp single reads were trimmed on the 3’ end, quality‐filtered and then split according to the 6‐bp unique barcode using custom Perl scripts (Miller et al., 2012). Sequences were dropped if the product of quality scores was below 80%. The barcode (6 bp) and restriction site residue (6 bp) were then removed from the 5’ end, resulting in a final sequence length of 80 bp. We used the software program Stacks version 1.29 (Catchen, Amores, Hohenlohe, Cresko, & Postlethwait, 2011; Catchen, Hohenlohe, Bassham, Amores, & Cresko, 2013) to identify orthologous sequences among *Zebrasoma* taxa. We first ran the program denovo_map.pl, which runs all three Stacks components in a pipeline (i.e., ustacks, cstacks, and sstacks). We set a minimum stack depth (−m) of three, a maximum of three mismatches per loci for each individual (−M), and allowed up to seven mismatches when building catalog loci (−n). We then ran the Stacks program *populations* to generate output files for input into downstream population genetics programs. Due to high coverage across individuals, we tested the minimum stack depth (−m) at six, seven, and eight, in population runs. We created a stringent dataset by setting −p at seven, which means both *Zebrasoma* species must retain the marker and with −r set to 100%, meaning that every individual in each species must retain the marker. Henceforth, a filtered 80‐bp sequence used in the subsequent analyses is called a locus.

### 2.5 Identifying outlier loci

We used two approaches to identify Φst outliers, which are commonly used to find loci under selection, but not without a number of potential pitfalls (Bierne, Roze, & Welch, 2013; Bierne, Welch, Loire, Bonhomme, & David, 2011; Fourcade, Chaput‐Bardy, Secondi, Fleurant, & Lemaire, 2013; Lotterhos & Whitlock, 2015). It is important that while outlier loci are likely to be under selection, our concern here was to identify outliers per se, without necessarily looking for evidence of selection. Values for Φst (AMOVA Fst) were computed by *Stacks* by comparing two populations, corresponding to individuals assigned to *Z. flavescens* and *Z. scopas* (without using individuals that were potentially hybrids). Then, we considered loci that were three standard deviations above the average Φst (AMOVA Fst), as obtained directly from the stacks population output (Weir, 1996). Instead, we used the Lositan workbench (Antao, Lopes, Lopes, Beja‐Pereira, & Luikart, 2008) that identifies loci based both on Fst and heterozygosity (generic Fdist approach). We found that the two methods had full overlap of loci, with Lositan being less stringent (more loci retained). In the long run, we decided to exclusively focus on the approach of keeping loci that were three standard deviations above the average Φst. All outlier loci were compared to GenBank entries with BLAST, where *E*‐values of 0.001 and below were kept and recorded (probability of obtaining the same result by chance <0.001). When matching sequences were found, protein‐coding genes were classified using KEGG assignments (Kanehisa et al., 2008; Ogata et al., 1999).

### 2.6 Structure analysis and species delimitations

A number of automated methods have been devised to identify species delimitations using multiple SNPs (Leaché et al., 2014; Zhang, Kapli, Pavlidis, & Stamatakis, 2013); however, those methods rely on phylogenetic assumptions that are not applicable here, where only two potential species are involved. Instead, assignment tests based on single nucleotide polymorphism datasets were run in the program Structure (Falush, Stephens, & Pritchard, 2007; Pritchard, Stephens, & Donnelly, 2000) with *K* = 4 populations (accounting for the two putative species *Z. flavescens* and *Z. scopas* in four populations, French Polynesia, Micronesia, Hawaii, and Japan). We compared a series of datasets. We used a dataset that included all loci. We then used datasets that corresponded to ranges of Φst values. The first dataset was based solely on outlier loci (those loci with Φst above three standard deviations from the mean). Subsequent datasets comprised loci whose Φst values had a range of one standard deviation, starting at the lower edge of the outlier loci. Therefore, the second dataset comprised loci whose Φst values ranged from three to two standard deviations from the mean, the third dataset from two to one standard deviation from the mean, a fourth dataset with loci from one standard deviation to the mean, and finally a fifth dataset that included all loci.

As our criteria for keeping loci were very stringent, our dataset did not include a large number of SNPs (just over 4,000 loci). We therefore also used a smaller dataset that only included those individuals with the highest number of SNPs to test whether our data were biased by lowering the total number of used loci (and they were not, as described below).

## 3 RESULTS

### 3.1 Phylogenetic reconstructions

Phylogenetic reconstructions based on neighbor‐joining and maximum likelihood methods resulted in identical topologies (Figure 2). As mentioned above, barcoding sequences (cytochrome c oxidase 1) suggest that *Zebrasoma scopas* is a paraphyletic assemblage of two species restricted to the Indian Ocean (one species) and Pacific Ocean (one species). Here, we only consider the Pacific Ocean species, the taxonomic status of *Z. scopas* being considered a separate issue not germane to this study. More important, CO1 sequences could not separate *Z. scopas* and *Z. flavescens* (Figure 2). As mentioned in Introduction, both findings have been published before, and our data, based on the latest available GenBank sequences, are consistent with those results. We therefore consider in this study that using samples of *Zebrasoma scopas* from the Pacific Ocean and *Z. flavescens* from Hawaii is a fair representation of the currently recognized species and the proper use of a natural group.

### 3.2 Single nucleotide polymorphisms

Restriction site‐associated DNA tag (RAD) libraries were created by individually barcoding 39 *Zebrasoma* individuals from Moorea, Micronesia, Japan, and Hawaii (Figure 1). Quality filtering of the raw data set left a remaining 36.1 million reads. For each individual, we identified an average of 58,983 stacks, of which an average 7540 were polymorphic, where each stack is comprised of filtered reads representing a potential locus. After specifying a depth of coverage of six and SNP presence in at least 80% of all individuals using the population script in *Stacks* (considering populations from Moorea, Micronesia, Hawaii, and Japan), we found a total of 1,961 loci, which corresponded to 5,193 SNPs.

Additional settings were used, including deeper depth coverage (up to 8), and using less samples with the highest read numbers (down to 22 samples, where sample numbers were equal for both putative species) to obtain balanced sampling and a larger number of loci and SNPs. In all cases, results were identical to the results presented here and conclusions remained unchanged (not shown).

### 3.3 Genetic structure between species and populations

**Figure 3:**
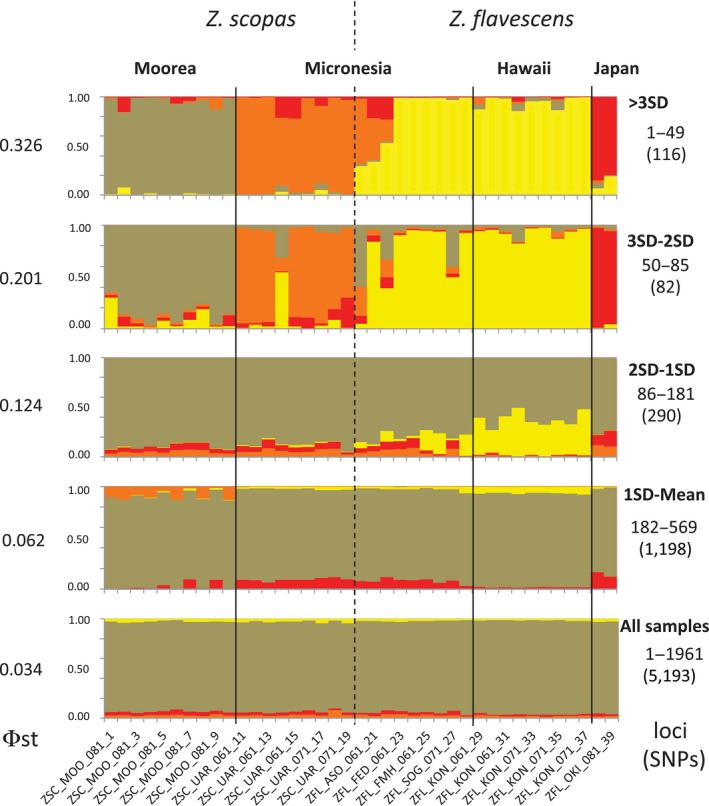
Structure analysis of *Zebrasoma scopas* and *Z. flavescens* samples using *K* = 4 (four populations). A total of 1961 loci corresponding to 5193 SNPs were used. Panels represent different datasets. The bottom panel corresponds to results drawn from all 1961 loci. The top panel corresponds to results drawn from using only outlier loci (three standard deviations above the mean of Φst). Subsequent panels move down from the top dataset by one standard deviation at a time. For example, the second panel from the top is based on loci that show a Φst value that is comprised between three and two standard deviations from the mean, the third is between one and one standard deviations from the mean, etc. A number of loci and SNPs used are shown on the right as numbers above and below (between parentheses), respectively. Values of Φst for the corresponding datasets are shown on the left. Population colors generally match colors used in Figure 1. Vertical lines separate samples by populations, and dashed lines separate individuals recognized as *Z. scopas* (left) and *Z. flavescens* (right). The first three individuals from Micronesia to the right of the dashed line were recognized as hybrids [Colour figure can be viewed at http://wileyonlinelibrary.com]

As mentioned above, *Zebrasoma scopas* and *Z. flavescens* are not distinguishable using mitochondrial barcoding (CO1) sequences. Results based on 1961 loci are consistent with these findings. *Structure* plots indicate that samples do not partition in distinct genetic clusters (Figure 3) and exhibit low levels of Φst between the two nominal species (Φst = 0.042). In the same way, we did not find evidence of population structure based on those 1961 loci (global Φst = 0.034, Figure 3).

### 3.4 Outlier loci

We identified 49 outlier loci in the dataset (2.5% of all loci), defined as exhibiting Φst values above three standard deviations above the mean. When running *Structure* with such outlier loci, a clear separation between *Z. scopas* and *Z. flavescens* was found, shown as brown‐orange and yellow clusters, respectively (Figure 3), which also corresponded to a higher pairwise Φst (Φst values of 0.356 and 0.520, see below). In addition, within *Z. scopas*, Moorea and Micronesia samples clustered in well‐defined groups (brown and orange clusters), and within *Z. flavescens,* samples from Japan separated from samples from Hawaii and Micronesia (red and yellow clusters). Differences between nominal species (*Z. scopas* – *Z. flavescens* – Φst = 0.438) were similar to differences between populations within species (Φst = 0.429). Overall, these findings resulted in a high global Φst value (Φst = 0.326).

Datasets corresponding to loci whose Φst values were gradually closer to the mean showed that delimitations between species and populations gradually disappeared (Figure 3). As expected, Φst values also gradually decrease as the datasets move away from the outlier loci (Figure 3). At the lower end of the Φst spectrum, we did not find evidence of loci under balancing selection (loci lower than three standard deviations from the mean).

### 3.5 Introgression and hybridization

Hybrids between the two species, with yellow/brown mottled coloration, have been reported at the region of range overlap (Figure 1) (Kuiter & Debelius, 2001; Randall, 2001). It would be impossible to identify hybrids based on all loci, as this dataset does not separate the two species in the first place. However, based on outlier loci (where species are distinguishable), we did not find evidence of introgression in *Z. flavescens* individuals from Hawaii. We also found that *Z. scopas* from Moorea hardly contain any genetic material assigned to *Z. flavescens* (Figure 3). In contrast*,* 29.3, 34.2, and 53.1% of genetic material identified as belonging to *Z. flavescens* was present in individuals from Micronesia identified as hybrids. The coloration of those individuals was brownish yellow (the expected color of hybrids).

The direction of introgression (greater abundance of *Z. scopas* genetic material than *Z. flavescens*) is also consistent with the much lower abundance of *Z. flavescens* compared to *Z. scopas* in Ulithi Atoll. Indeed, the relative abundance of *Z. scopas, Z. flavescens,* and their hybrids was estimated at 90.9%, 5.6%, and 3.5%, respectively (authors’ unpublished data recorded from 2013 to 2016, based on 102 visual transects over 32 sites, total number of *Zebrasoma* individuals = 428). A pattern of directional introgression, as was observed here, that may be predicted by skewed abundances of parental species, is not unusual, as was reported in several other coral reef fish species (Crow et al., 2007; Harrison et al., 2017; Montanari, Hobbs, Pratchett, Bay, & Van Herwerden, 2017; Yaakub, Bellwood, Herwerden, & Walsh, 2006).

### 3.6 Outlier loci GenBank matches

Of 49 outlier loci, approximately half of the sequences (24 sequences, 49%) matched GenBank entries. All 24 matches corresponded to fish sequences, 20 sequences matched protein‐coding regions and the remaining four sequences matched unannotated fish genome sequences (Supporting Information Table S1).

Protein‐coding regions were analyzed using the program *Kegg Koala* and we found that almost half of the sequences (49%) clustered in only three functional categories: genetic information processing (21%) (transcription, translation, replication, repair); environmental information processing (15%) (membrane transport, signal transduction); and organismal systems (13%) (immune system, endocrine system, nervous system). The large number of protein‐coding genes (almost half of the outlier loci) is larger than expected. This was tested using 20 randomly selected datasets of 49 loci (the same number as the actual outliers) and determining how many protein‐coding genes were comprised in the new datasets. The highest number of protein‐coding genes in random datasets was 12 (as opposed to 24 in the actual outliers), and the number obtained with the actual outlier loci (24) was significantly different than what was found in randomly chosen datasets (*t* test, *p* < 0.01).

## 4 DISCUSSION

Defining species delimitations has been a challenge for over a century (Coyne & Orr, 2004). The original goal of Darwinian principles was to classify organisms according to their descent, which translates into genetic lineages traceable by their molecular markers (Cracraft, 1983; Wiley, 1981). Given enough time, all markers show the same picture, due to sorting processes (Avise, 2004). However, full concordance of markers is only apparent in those lineages that diverged a very long time ago (on average, 4Ne generations are necessary for sorting to occur (Avise, 2000, 2004)), which is not the case for incipient sister taxa (e.g., Egger, Koblmüller, Sturmbauer, & Sefc, 2007; Noor & Feder, 2006).

The idea of accepting species delimitations when congruence was found among several molecular markers emerged when scientists moved from using a few mitochondrial markers to a combination of mitochondrial and nuclear markers (e.g., Bernardi et al., 1993). The practicality of the approach was reasonable at the time; however, with the current use of thousands of genomic markers, the picture became more complex. Recent work has shown that genomic islands of diversity may distinguish divergent populations, thus negating, even on a theoretical standpoint, the possibility of congruence among genomic regions. These islands may either be the cause of speciation (genomic islands of speciation) or the result of local adaptation in populations that diverged, usually in allopatry. The concept of genomic islands of speciation has the distinct advantage of providing both an explanation for the speciation event (the emergence of the islands by divergent selection) and a way to define species (the composition of the islands differs in separate populations and therefore defines them genetically, and can be visualized in different ways, such as with structure plots or with pairwise differentiation, Fst, plots). In particular, their role in driving speciation events is very compelling when present in sympatric species, where other reasonable explanations for speciation mechanisms are unlikely (Marques et al., 2016).

In this study, we found that mitochondrial markers and most nuclear genomic markers do not delineate *Zebrasoma scopas* and *Z. flavescens*, yet outlier markers do. These markers are consistent with the presence of genomic islands of divergence. In addition, evidence presented here (the prevalence of protein‐coding genes) points toward the idea that these outliers are likely to be undergoing diverging selection.

An interesting case was presented for two other surgeonfish sister species, *Acanthurus olivaceus,* which has a very broad geographic range, and *A. reversus,* a Marquesas islands endemic (Gaither et al., 2015). Similar to our system, mitochondrial markers and thousands of neutral RAD markers do not separate the two species. In contrast, outlier loci separate the widespread *A. olivaceus* from the Marquesan *A. reversus*. It is important that the isolated Hawaiian population of *A. olivaceus* could not be defined by outlier loci, thus suggesting that local adaptation in the Marquesan environment, rather than simple genetic drift, was proposed to be responsible for the observed patterns (Gaither et al., 2015). A simple interpretation of this situation is that genomic islands of divergence are present in the system, and these are the consequence of isolation and local adaptation rather than divergent selection driving speciation.

While the final genomic signature is very similar, teasing out the alternative scenarios that genomic islands of divergence are the cause of speciation or the consequence of isolation and adaptation is important to understand the fundamental mechanisms of speciation and ultimately the generation and maintenance of biodiversity. The ecological and biogeographic context of the *Zebrasoma flavescens–Z. scopas* complex shows strong similarities with the *Acanthurus olivaceus–A. reversus* system, and in that respect, the emergence of genomic islands of diversity *after* the isolation events seems more likely that their emergence as a mechanism of speciation.

Besides very slight modal morphological differences, there are very obvious coloration differences between species (*Zebrasoma scopas* is solid brown and *Z. flavescens* is solid yellow) (Randall, 2001) that allow for easy and rapid identification, and sorting of individuals in two distinct groups. While the absence of reciprocally monophyletic mitochondrial clades would traditionally be seen as a lack of support for current taxonomy (i.e., the separation of *Z. flavescens* and *Z. scopas* as bona fide species) one must account for the fact that sequencing specific molecular markers (outliers) result in the very same groupings obtained by sorting the actual fish for coloration. These modest levels of morphological difference and genetic divergence are, in fact, commonplace in cichlids where hundreds of species are described (Wagner et al., 2013).

The genetic differentiation levels, again based on outlier loci, of the Moorea/Micronesia populations of *Z. scopas*, and of the Hawaii/Japan populations of *Z. flavescens*, which are similar to the divergence between the nominal species bring up additional questions of population delimitations and the role of the environment in the early stages of speciation. Taxonomic debates as to the validity of species and population nomenclature are interesting but not the focus of this study. Our goal is neither to invalidate nor validate the status of *Zebrasoma* species, but rather to investigate the dynamics of genomic divergence between groups of *Zebrasoma* individuals.

The advent of massive genomic sequencing uniquely affords to explore speciation mechanisms in great detail, yet we must also face the reality of the complexity of species delimitations (Bernardi, 2013). Here, we show that the use of outlier loci provides unambiguous partition of individuals that have independently been recognized as species. These loci are likely to have evolved under different environments, as shown by the type of outliers that were found. In this study, 15% of outliers are related to environmental conditions; in the Marquesan *Acanthurus* study*,* opsin Rh2, a gene involved with color vision, was found among the outliers. By being locally adapted, individuals are indeed different in each region, as predicted by several models of ecological speciation in coral reef fishes (Rocha & Bowen, 2008). While speciation via local adaptation has been recognized for a long time, here we show that the number of loci involved is relatively small, but nevertheless sufficient to clearly distinguish genetic entities, and this type of speciation that can easily be overseen unless thousands of loci are analyzed must be reckoned with.

## CONFLICT OF INTEREST

None declared.

## AUTHOR CONTRIBUTIONS

All authors designed the experiment and revised the manuscript. GB did the molecular work and wrote the first draft of the manuscript.

## DATA ACCESSIBILITY

All Fastq sequence files are available from GenBank at the National Center for Biotechnology Information short‐read archive database (accession number: SRP139089). Associated metadata are also available at GeOMe (GUID http://n2t.net/ark:/21547/BAf2) (Deck et al., 2017).
