# Supplementary material for: Genomic islands of divergence in the Yellow Tang and the Brushtail Tang Surgeonfishes
Source: Ecol Evol. 2018 Aug 2;8(17):8676–85. doi: 10.1002/ece3.4417 (PMC6157655; doi:10.1002/ece3.4417)
Supplement: Supplementary file 1 [file ECE3-8-8676-s001.pdf]

Table S1: GenBank matches with outlier loci. Outlier loci (49) are ranked by levels of Phist (second column).

Their locus ID (stacks output) is in column 3, their sequence in column 4, the GenBank match accession number (column 5) and the description of the locus in column 6 when applicable.

	Phist	Locus ID	sequence	GenBank accession	E-value	Locus
1	0.775	29834	ACTGGTGGCTGGTGTACTGGTGAGTGTTTTTTGCATTGTTTGATAGGTAAATGCACTGCTGTGGCTCACTTTTCTGTGTA			
2	0.7175	11724	ACCTCCTGCGCCTCCTTCCACGATGAAACAGCCTCTTCCAGGAGGCGCTCTGCCTCAGCACGCCTGTTCTCCACCTCCAG	XM_019274056.1	2 e^-21	Larimichthys crocea pleckstrin homology domain-containing family O member 1-A (LOC104936844)
3	0.6475	15560	AGGCGGAGTCTCTGCTCATTGTTGAACCTCTCCACTGCTGCCCAGAACCACCGGATCACAATGTGGTTGTCGTGATAACC	XM_020657487.1	3 e^-27	Labrus bergylta E3 ubiquitin-protein ligase HECW2-like (LOC110001920)
4	0.5924	53705	AGCTGTGTCTGCTGGCAGAGGAGAAGGTGGCAGCTGCCGGAGGCTGCCCTGCAGAGCTGCTGGCTTGTCCCTCCGCTTCG	XM_019253842.1	6 e^-23	Larimichthys crocea ankyrin repeat and MYND domain-containing protein 2-like (LOC104931086)
5	0.5605	35824	TGAGCTGGTTTCAGTGTCTCTGAATGAAGTTGTGCGGCTGCAGTGTCCCGCAGCATCACGGCTGTCCCAGCAGCTGTGGG	XM_019254908.1	2 e^-23	Larimichthys crocea semaphorin-4A (LOC104933068)
6	0.5161	18186	GTGGTGTCACCAACTCCTTCAGACCCAGTAGCTGTTCCCCTCTCTCTGAATGTAGCAAGTGACTTCTGCGGATCGTCTGC			
7	0.4984	35261	CTAATCAGGTCACACACACAGTGATCTGCTATAGCTGAGTCCATAATAGTAAAATGACATAGACCAATGCTGTTTACATG			
8	0.4491	14819	TGTAGCGGCACTTTGACTTGGCTGCTTTCTGCAACTCAGAGCTTTTCTCAGGCCGTGATCACATAGGGTGTGTTTTTGCT			
9	0.4341	29737	GTCTTTTGGTACCAGAGGAGAGAGGAATGATTTGATTTCTTACACTACTTTCAATTCCACCCAGCCGGTCTTCTAAAAAT			
10	0.426	5472	AGCAGCTGACGGCCGACGGCTTTCCAGTGACCGTCAACGCCGTGGACCCTGGCATGGTGGACACGGCGCTGTACGACAAC	XM_019264920.1	7 e^-22	Larimichthys crocea dehydrogenase/reductase SDR family member on chromosome X-like (LOC109139865)
11	0.3973	44763	CCGACAGGGTCAGCTATTACCTGTGCCGGGGCAGAAACGGAATACCTGAGCTCGCCACTTCCCTTTTTGACGTGACGCCG	XM_019271944.1	2 e^-21	Larimichthys crocea phosphodiesterase 6C (pde6c)
12	0.3717	45022	TGCAAGCGCTACTCCAGACAGCAGAATGATTCGGGACACAAGGACACATCTGTCCACAGTGGTAAACAGAGAAAAAATCA	XM_018695518.1	1 e^-24	Lates calcarifer sclerostin domain-containing protein 1-like (LOC108896394)
13	0.3388	14523	TGGAACTAGTCAAACTCCCCTGGAAGAGCTTGTTCTAAAGAAGATCCAGGATGAAGTCTTCAAACAGGATCATGGCAAAG			
14	0.3302	16122	GCAGCCAAACTGCCTCCTGATGAAGTCCTATCAGTCCTCTGCTGGTCTGTTGGCTAGTTTTTATGATGTTGCCTCCAGCT			
15	0.3022	8815	AGATGAACCTGCTCCTGGTGCATGAGGACGTAAATGAGGTTAACGTCTGCAACTTTACGTCTGGCAAACTTTGAGTTTGA			
16	0.2926	5668	GTTACAGGCAGAGCAGAGAGTTCATCATCACCCAGAATCCTTTGCCCGGCACCATAAAAGACTTCTGGAGGATGATATGG	XM_023268706.1	2 e^-22	Amphiprion ocellaris receptor-type tyrosine-protein phosphatase zeta-like (LOC111567526)
17	0.2915	34093	AGGATGCCGTACTGCCGGATTTTGTGTGATTTCAGCCAAAGTGCCTCATCCTCTCCTCTGCTGGGTATGTATGCATATTT			
18	0.2914	15659	CGGTACAAGCTTGGCTGCAAGCTTCTCTCAATCCATCTCCACTACCATCCCTCTTTATTTTCAACTGGGGTGGCTCACTG	FQ310507.3	2 e^-22	Dicentrarchus labrax chromosome sequence corresponding to linkage group 1
19	0.2883	42327	TGACATGAAGTATTGGCACAGTCTGTCTCGTTGTTTCTCAGCTTCAACGGAAGGGTTTCTTGCTCTCCCCACCAGTGCAG	XM_015600903.2	6 e^-4	Astyanax mexicanus uncharacterized LOC107196892 (LOC107196892)
20	0.2837	37909	ACGTCGTTCTCACATCAAAACATCACATGAGGGGAGTAACTTTTGAGTATAAAATCCCTCCAGCAGAGTTTCAGAGGTTT			
21	0.2819	49540	CACCAAATAACATTAAAAATAACGAGTGATACAACTTCTTTCTCTTCAGTTTCATTTCTTCCGCACTCTGGGTCAAAGAA			
22	0.279	15134	CTGCGAGCTGACCTTTAACCTCAACACACAGCACCATTAACACAACCATGCATGTAACAGGTGACGATGGCTCATGTGGG			
23	0.2768	28898	TGCTCAAGTTTCCCCCAACAGCATGCACATATAGGTAAATTCTCCTGTCTGTGCCCTTGAACACAGCACTGCTCAGAGCT			
24	0.2755	48045	CTGACCGATGCCAAGGGATGGTTTGGATCTGAAAAGTCACTCTGACTCTCCACCTGTGTCCTGTTACATTCCCAGAGGAC			
25	0.2636	6349	TTGAGTCTCTGATAAGCGGTTTATACCACGGGCCACGGCGCCCGAAGCATTTTTGCATTGTCTTGTCTTTGCTGACAAGA			
26	0.2592	3520	ACACCGGCAAGACAGGCATCTGTTATATTTGTTCTAATGTTTTGAATCTGGTCCTGGGTTACTGCCAGGGAGTCCTGAAT			
27	0.2585	43051	CAGAGGAGGGTGTTCATCCTGTGAGCCCAGCTGTTCCCCTCACACACTCGGAGGAAGATGGCTGCTGGGTTGCTGTTGCT	FQ310508.3	6 e^-16	Dicentrarchus labrax chromosome sequence corresponding to linkage group 18
28	0.2578	24979	GCAGAAAGAGGCTTGTGACAGGCTTTGACAGGTACGGCATGGTGGCAGACCTCCCAGCTGTCAAACAAACAGACTGACTA	DQ481663.1	2 e^-9	Takifugu rubripes HoxAa gene cluster
29	0.2521	27158	ACCCCGCATTCCAGGGCGTCCTGGATAACCGGGAGAGCCGCTCCTGCCGGGGTAGCCTGTAGTCCCCATAGAGCCTTTGG	XM_018673612.1	4 e^-18	Lates calcarifer collagen alpha-1(XXI) chain-like (LOC108881541)
30	0.2493	22180	TCTTAGAACGTGACTTGGAGTTGCGTCATCGTGATGAAAGACGTGTGAGGTTAGAGGTGACTGTTAATCCATTTCAGGTG	XM_018697558.1	3 e^-8	Lates calcarifer synaptotagmin like 1 (sytl1), transcript variant X3, mRNA
31	0.2416	26853	TTGTAGTGGAGCTCATCACCTCTGCCAAAGGACTCTTCTCTTTGCTCAACAGGTTTGAAAAGAGCGAGTTATCTAACCGT	XM_018663698.1	8 e^-15	Lates calcarifer connector enhancer of kinase suppressor of Ras 1 (cnksr1)
32	0.2407	37693	TCCAGAGCAGTGAGCTGCTTTCCCTTCTCTCCTGAGTTTCGTTTATACTCCTCGATTGTCCAGGACGGCTGGCCACGCTT	XM_022744056.1	7 e^-22	Seriola dumerili KIAA1024 ortholog (kiaa1024)
33	0.2406	16140	TATTATGCAGGAATATTGGGCGGCAAGTGGCTTGCAGTCACAGATCCAGACTTTACAGGTGTGGCAAAGATGCTTGCAGG	XM_022757641.1	6 e^-4	Seriola dumerili BRI3 binding protein (bri3bp)
34	0.2397	580	AACCTTCTCCAGAAAAGAGATATGGGAGCAGAGAGCACACTATGCACTTCAGTGTGCAGAGGTTAACTTGAGGATATTTA			
35	0.2397	22458	GTGGAGAGTTTTTGAGAAACCAGGCACAGAGTCTGGATGAGAGTGACAGTGGTGGAAATATCACAGAGGATAGTCAGAGA			
36	0.2397	71973	TCCCCCTCCCCTGATACTGACGTCCAAATAAATCTCTAAGCCCCCTCTACCGACGATGCAACATGAAAGAGGGCACAGCC			
37	0.238	8981	GTACAAGGAGGTTTCATGTGGGTACGAAGCACCGTTGTCACTTCGGCAGAAATCTCGACAAAGCAGTTCGTGATAGCCCC			
38	0.238	12181	GCAGCACCACTGAGCCGTCCCTGGAGGAGAAGTACCGGCTCCTGGGACCAGCTGACACACCTCTGGGGCACATGCCTGAT	XM_020634254.1	6 e^-29	Labrus bergylta transmembrane protein 266-like (LOC109983921)
39	0.238	20184	CGGCTGGCGACCAGGACAAAGCTGGCAAAGAACAGGGCACTGCAGATCAGTGGTATGGGCCCGGACCAAGCCTCCGCCCC	XM_023290912.1	1 e^-31	Amphiprion ocellaris protein PRRC2A-like (LOC111582296)
40	0.238	54181	TTTGTCAAAGTAAACCTTTTGTTTCTGCAGGCTGCCTGCATAGTGTCTTGATTTTCTCCAGGCATCTGAAATGTAACAAC			
41	0.2376	13577	TGAAGCCACATAGTGACAATGCCAAAATTTTATTACTGGATTGTATTGCTGACATTAAGCAGTGGTTGGCCCAAAATTTT	XM_019254115.1	2 e^-4	Larimichthys crocea RNA-directed DNA polymerase from mobile element jockey-like (LOC109137022)
42	0.2376	15998	GTAATGAGATCGGATCCATCATGGACCCCACCAGGTTCTCTCGTAACTACAAGTTTGGATCTATTGAGGGCAACATGATC	XM_023267030.1	4 e^-19	Amphiprion ocellaris apolipoprotein B-100-like (LOC111566442)
43	0.2367	42593	GTGGATGATAAATCTCTTAAGAACAAAGTTTATGTACCAGCAGATTAGATCCACTGCTTCATATTCACAGTGTGTCAGCC	XM_005948727.2	1 e^-19	Haplochromis burtoni purinergic receptor P2Y, G-protein coupled, 1 (p2ry1)
44	0.2367	52221	AGCAGATGACTTTGTTAGGCTGCTGTCACAAAGTGAACCGGGTTTCAGCACACCAGCTGACAAGACCGCTTTGATGATTC			
45	0.2364	19565	CAGCGGCAGCAGCAGTAATAACGGCTCTCCATCTCCCAGCCCCATTTAACTGACTGATGCTTTTTGACATGTCGGAGCAA	CP020801.1	2 e^-23	Oryzias latipes strain HNI chromosome 23
46	0.2356	4192	CTCCGCCTCCTGCATGGTAACTCCACCCTGACACTGATGCACCTGCACAGTGGTGTTGGTGCTTGACAAACTGTTCCCTC			
47	0.2356	5409	TCAAACATCAGAGGTGAAGGTTCGGCACAGATGTTATCAGCTGGCTGCTTGTGCCAAGCTGACGACACTCGATGCTTGAA			
48	0.2356	18936	ACAGAGCCGTCTCTTGGAGTTTCGGGCTCTCGGCGCCTGCACCCTGCTCCACATGGTCAGACTGCTGTACTCTGGAGAGA	XM_018679929.1	7 e^-22	Lates calcarifer BTB domain containing 18 (btbd18), transcript variant X2
49	0.2356	40384	ACCGCAGTGGCTGCTGTCTGTCAGTACTCCTCTGTAGCTAATCGATGGATTAAAGATGCTGATGAGACTCAGAGAGAAAG			
